# Sensitivity and specificity of single IgA and IgG antibody concentrations for early diagnosis of pertussis in adults: an evaluation for outbreak management in public health practice

**DOI:** 10.1186/1471-2334-7-53

**Published:** 2007-06-06

**Authors:** Paul LJM Mertens, Frans S Stals, Ewout W Steyerberg, Jan H Richardus

**Affiliations:** 1 Department of Public Health, Erasmus MC, University Medical Center Rotterdam, 3000 CA Rotterdam, The Netherlands; 2 Municipal Health Service Rotterdam Rijnmond, Rotterdam, The Netherlands; 3 Laurentius Hospital, Roermond, The Netherlands

## Abstract

**Background:**

An accurate, practical laboratory test is needed to confirm clinical diagnosis of pertussis in adults during the first 3 symptomatic weeks, when treatment is effective and transmission can be interrupted.

**Methods:**

The sensitivity and specificity of single IgA and IgG levels were assessed in a cohort study of a pertussis epidemic in 99 adults in a closed community. Sensitivities were assessed in the sera of 46 laboratory confirmed clinical pertussis cases during the first 3 weeks. Specificities were calculated in sera of 35 asymptomatic controls without clinical symptoms or laboratory confirmed infections from the same community (internal controls). We compared these specificities with the specificities of single IgA and IgG levels in 4275 external controls from a cross-section of the general Dutch population aged 21–79 years who had not coughed for more than 2 weeks in the past year, and without pertussis diagnoses. The study was done in the Netherlands when whole-cell pertussis vaccine was used in the national vaccination programme.

**Results:**

Levels of 24 U/ml for IgA and 27 U/ml for IgG gave sensitivities of 100% and 75%, respectively, in the first 2 weeks, 100% in the third week, and 97% after the fourth week. The levels were reached within 2 days after onset of increase, and remained above these levels for roughly 7.2 and 5.1 months, respectively. Specificity was 82% for IgA and 89% for IgG in the internal controls and 90% in the external controls, respectively.

**Conclusion:**

We suggest levels of 24 U/ml for IgA level and 27 U/ml (= 27 International Units (IU)/ml) for IgG as sensitive, specific, and practical for laboratory confirmation of clinical pertussis in adults in the first 3 weeks of outbreak management.

## Background

Pertussis is a bacterial infection caused by *Bordetella pertussis*. Despite the introduction of mass vaccinations in the Netherlands in 1952 and other countries, pertussis is still an endemic disease with regular epidemic outbreaks.[1-5] In unvaccinated populations, pertussis morbidity and mortality occur predominantly in those 15 years or younger.[6] In vaccinated populations, pertussis is more likely to be found in older children and adults.[7-13] Circulation of *Bordetella pertussis *in vaccinated children, adolescents, and adults plays an important role in the continuing transmission of the pathogen to infants too young to be vaccinated, in whom the disease is the most severe and sometimes fatal.[14-19]

Pertussis is most infectious in its prodromic and early clinical stages. Effective management in unvaccinated infants and during outbreaks requires early diagnosis and treatment of cases, accompanied by antibiotic prophylaxis of contacts.[20,21] There is no evidence of benefit from chemoprophylaxis given more than 21 days after the onset of the primary case.[22,23] Therefore, antibiotic management of pertussis should be initiated promptly to minimize secondary spread.[24] For the improvement of clinical diagnosis and pertussis surveillance, rapid, easily accessible, highly sensitive and specific laboratory methods are needed to diagnose pertussis in the first 3 weeks.[25] The validity of methods for pertussis diagnosis depends on the time of initiation. Early in the disease, culture and PCR can be used. Culture of *B. pertussis *is highly specific, but laborious and insensitive.[26] The yield progressively decreases during disease[27] and is low after 2 weeks and antibiotic use.[28] The sensitivity of PCR is superior, but rapidly decreases with increasing duration of the disease and patient age.[29,30] Serology is a good alternative.[29,31,32] In this study, we assessed the sensitivity and specificity of single IgA and IgG antibody levels for laboratory confirmation of clinical pertussis in adults during the first 3 weeks of symptoms. The study was done in the Netherlands when whole-cell pertussis vaccine was used in the national vaccination programme.

## Methods

### Study population

The clinical and laboratory outcomes of a pertussis epidemic in a religious convent, which has been described previously, was used as a sample population to evaluate single IgA and IgG levels after other causes for an outbreak of coughing were excluded.[12] In short, the epidemic was reported to the Municipal Public Health Service (MPHS) in its 9^th ^week for outbreak management, at which time the study began. The convent was a nursing home for 75 elderly resident nuns supported by 24 non-resident personnel. The personnel consisted of 3 males and 21 females. The mean age was 75 years (range 55–94 years) for the nuns and 27 years (range 21–46 years) for the personnel. All of the nuns and 5 of 24 non-resident personnel had never been vaccinated against pertussis. The nuns and staff shared common social and religious activities, and shared meals in the dining room. Pertussis cases were cared for in the convent by nuns and personnel. Retrospective interviews based on structured questionnaires were conducted to obtain information about the onset of characteristic clinical symptoms in the first 9 weeks of the epidemic. The emergence and duration of the clinical manifestations were prospectively monitored daily from week 9 up to week 13, the period that the epidemic persisted with new cases. After week 13, cases were monitored weekly until symptoms subsided. Two nasopharyngeal swabs were obtained from each individual at weeks 9 and 13 of the epidemic, one for culture and one for PCR. At weeks 9, 13, and 60 serum samples were obtained to determine IgA and IgG antibody levels.

### Definitions

*Laboratory confirmed B. pertussis infection *was defined as one of the laboratory findings: 1) *B. pertussis *strain isolated from the nasopharynx; 2) reactive PCR; 3) significant (at least 4-fold) increases or decreases[33,34] of IgA or IgG antibody levels between at least one pair of serum samples obtained at weeks 9, 13, and 60 of the epidemic; and 4) IgG level ≥ 100 U/ml at weeks 9 or 13 of the epidemic[35] (equivalent to 125 International Units (IU)/ml).[12] We defined *clinical pertussis *as a persistent cough and its duration was divided in "7–13 days" and "at least 14 days".[36]* Pertussis cases *had both laboratory confirmed *B. pertussis *infection and clinical pertussis. The index case was the first pertussis case in the epidemic. *Pre-epidemic cough *was defined as a cough that occurred before the emergence of the index case. *Internal controls *had no laboratory confirmed *B. pertussis *infection and were asymptomatic residents or non-resident personnel, i.e. they had no pre-epidemic cough nor clinical pertussis.

*Sensitivities *of different IgA and IgG levels were calculated as the proportion of serum samples with a positive test result. The calculations were done in samples obtained from pertussis cases who had coughed for at least one day during their period of clinical pertussis. *Specificities* of different IgA and IgG levels were calculated as the proportion of serum samples with a negative test result. These calculations were done in samples obtained from the internal controls. Specificities were also calculated in 4275 external controls from a cross-section of the general Dutch population in the same age group (21–79 years) as the convent population. Control subjects reported in a structured questionnaire to have not coughed for more than 2 weeks in the past year, nor to have had a physician-diagnosed pertussis.[35,37] From the external controls, specificities of different IgA and IgG levels were calculated as the proportion of the 4275 serum samples with a negative test result.

### The duration of waxing and waning of IgA and IgG concentrations

To estimate the time period after which single IgA and IgG concentrations can be reused as a diagnostic test for a subsequent pertussis infection, we assessed the duration (in days) of waxing and waning of IgA and IgG concentrations after a *B. pertussis *infection. Therefore we first calculated the geometrical mean concentration (GMC, U/ml) over the highest IgA and IgG levels obtained in week 9 and 13 of the epidemic for all subjects with a significant increase or decrease of antibody level. In these subjects we then calculated the mean rate of increase (expressed as U/ml/day) towards the GMC and the mean rate of decrease from the GMC downwards. This was done for IgA and IgG separately.

### Laboratory methods

In the two populations, the serological laboratory investigation of pertussis specific IgA and IgG antibodies was performed by enzyme-linked immunosorbent assay (ELISA) at the National Institute for Public Health and the Environment (RIVM), the Netherlands, as described previously.[12,31,35,38] For IgA class antibody detection, a crude cell-wall preparation of *B. pertussis *was used. For IgG class antibody detection, purified pertussis toxin was used. Antibody binding activities were quantitatively expressed in 'local' units per milliliter (U/ml). IgG can be converted to FDA international units (IU) non-linearly by the formula Log_10_(U/ml) = 0.2174 + 0.8475log_10_(IU/ml).[39,40] The detection limit of the assays was 5 U/ml.[35] In our evaluation of sensitivities and specificities of single IgA and IgG levels, we focused on levels at least 4 times the detection limit. Culture and PCR were processed as described previously.[12,30,41]

## Results

### Convent population

The convent population consisted of 75 nuns and 24 personnel. All 99 individuals participated in the study. The pertussis epidemic started in week 1 with the emergence of the first case with laboratory confirmed pertussis infection. The last case was detected in week 13. During the study, 6 residents died resulting in 99, 99, and 93 study subjects in weeks 9, 13, and 60 of the epidemic, respectively.

### Clinical outcome

Retrospective evaluation of weeks 1–9 of the epidemic revealed 2 subjects with a pre-epidemic cough, 3 subjects with clinical pertussis of 7–13 days duration, and 41 with clinical pertussis coughing 14 days or more. Prospective evaluation in weeks 9–13 of the epidemic revealed another 6 subjects with clinical pertussis coughing 14 days or more and 47 with no cough (Table 1). Clinical pertussis persisted for a mean period of 69 days (range: 7 – 268 days).

**Table 1:** Outcome of clinical and laboratory investigations in absolute numbers in the convent population (N = 99)

**Outcome of laboratory tests for pertussis infection**	**Clinical Outcome**				**Total**
		
	**Clinical pertussis coughing > 14 days**	**Clinical pertussis coughing 7–13 days**	**Pre-epidemic cough **	**No cough **	
	** (n = 47)**^c^	** (n = 3)**^c^	**(n = 2)**	**(n = 47)**^e^	**(N = 99)**
**Laboratory confirmed pertussis infection:**	45^b^	1^b^	0	12^f^	58
IgA or IgG 4-fold increase or decrease between week 9, 13 and 60 of the epidemic	41	1	0	9	51
IgG ≥ 100 U/ml at week 9 or 13 of the epidemic, no IgA or IgG 4-fold increase or decrease	4	0	0	3	7
*Details of laboratory confirmed pertussis findings:*					
*- IgG ≥ 100 U/ml*	*41*	*0*	*0*	*6*	*47*
*- IgA 4-fold increase*	*8*	*1*	*0*	*2*	*11*
*- IgA 4-fold increase or decrease*	*22*	*1*	*0*	*5*	*28*
*- IgG 4-fold increase*	*6*	*1*	*0*	*2*	*9*
*- IgG 4-fold increase or decrease*	*39*	*1*	*0*	*9*	*49*
*- PCR or culture positive*^a^	*5*	*0*	*0*	*0*	*5*

**No laboratory confirmed pertussis infection**	2	2	2	35^d^	41

^a ^All culture and PCR positive subjects had a significant increase of IgA or IgG as well

^b ^Pertussis cases for sensitivity calculation

^c ^Subjects for calculations of spectrum bias in sensitivity

^d ^Internal controls for specificity calculation

^e ^Subjects with no cough for calculations of spectrum bias in specificity

^f ^Subjects with asymptomatic pertussis infection

### Laboratory outcome

At weeks 9, 13, and 60 of the epidemic 94, 97, and 85 serum samples, respectively, were obtained. Three samples were collected from 80 subjects, and 2 from 17 subjects. One serum sample was obtained from one resident with, and one resident without clinical pertussis. Of the 99 subjects, 51 showed a significant increase or decrease in IgA or IgG between week 9, 13 and 60 of the epidemic, 5 of whom also had either a positive PCR or culture. Additionally, 7 subjects had a single IgG level of ≥ 100 U/ml at week 9 or 13 of the epidemic, with no further significant increase or decrease of IgA or IgG. This resulted in 58 subjects with, and 41 without laboratory confirmed pertussis infection (Table 1).

### Pertussis cases for sensitivity and internal controls for specificity calculations

Table 1 shows the source data for the sensitivity calculations, the 46 pertussis cases (45 cases with clinical pertussis coughing 14 days or more and 1 case coughing 11 days, all with laboratory confirmed pertussis) and the 35 internal controls who had no cough and no laboratory confirmed pertussis infection. The mean age of the cases and internal controls was 77 years (range: 21–86 years)  and 56 years (range: 22–91 years), respectively.

The levels of IgA and IgG obtained from the 46 pertussis cases and the 35 internal controls are shown in Table 2. The geometrical mean concentration (GMC) of IgA and IgG in the 46 pertussis cases was significantly higher (p < 0.001) in all sampling weeks compared to the 35 internal and 4275 external controls.

**Table 2:** The Geometrical Mean Concentration (GMC), with 95% CI, of serum samples from pertussis cases and from internal controls at week 9, 13 and 60 of the epidemic, and of the serum samples from the external controls (n = 4275)

**Subjects and time of sampling during the pertussis epidemic**	**Number of samples**	**IgA**		**IgG**	
		
		**GMC**	**95% CI**	**GMC**	**95% CI**
Cases (n = 46)					
- week 9	43	123	75 – 202	252	119 – 535
- week 13	46	172	121 – 244	355	235 – 537
- week 60	40	60	40 – 89	34	22 – 53

Internal controls (n = 35)					
- week 9	34	9.4	6.4 – 13.7	6.0	3.9 – 9.3
- week 13	34	7.8	5.2 – 11.6	3.8	2.4 – 6.1
- week 60	29	6.6	4.4 – 9.9	2.8	1.8 – 4.2
- week 9, 13 and 60 combined	97	7.9	6.3 – 9.9	4.1	3.1 – 5.3

External controls (n = 4275)	4275	8.5	7.9 – 9.1	7.4	6.7 – 8.3

### Course in time of IgA and IgG in pertussis cases

**Figure 1:**
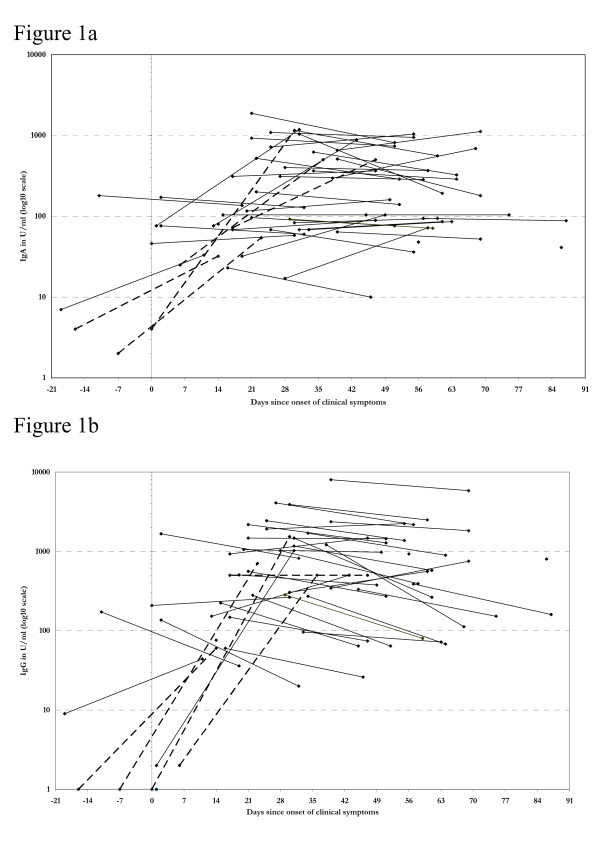
**The course of IgA and IgG**. The course of IgA (figure 1a) and IgG (figure 1b) levels obtained in all 89 serum samples from the 46 pertussis cases obtained in week 9 and 13 of the pertussis epidemic in the convent population (n = 99). Pertussis cases had a laboratory confirmed *B. pertussis *infection and a clinical pertussis (see methods). The levels are related to the first day of cough of the pertussis cases. Six serum samples were obtained from cases before they started coughing and 83 samples were obtained from cases who had been coughing for between 1 and 87 days. Lines connect samples obtained from one subject. Dotted lines are from the 5 subjects with a positive culture or PCR.

Figure 1 shows the longitudinal courses of the 89 IgA and IgG concentrations obtained from the 46 pertussis cases in weeks 9 and 13 of the epidemic, as related to the onset (day 0) of the clinical period (coughing). Six pre-clinical and 83 clinical serum samples were obtained from the 46 pertussis cases. The earliest serum sample was obtained 19 days before, and the latest one 87 days after the onset of cough.

The general pattern of IgA (Figure 1a) shows a more consistent increasing and decreasing pattern compared to IgG (Figure 1b). IgG concentrations reached higher levels than IgA concentrations and tended to decrease more rapidly to relatively lower levels earlier in the clinical period compared with IgA. From 3 of the 5 PCR or culture positive cases (dotted lines in the figures), the first serum samples were obtained before the onset of the cough. These 3 cases showed at least 4-fold increases of IgA and IgG levels, starting from below 5 U/ml.

### Sensitivity and specificity of single IgA and IgG levels for estimating optimal cut-off values

**Figure 2:**
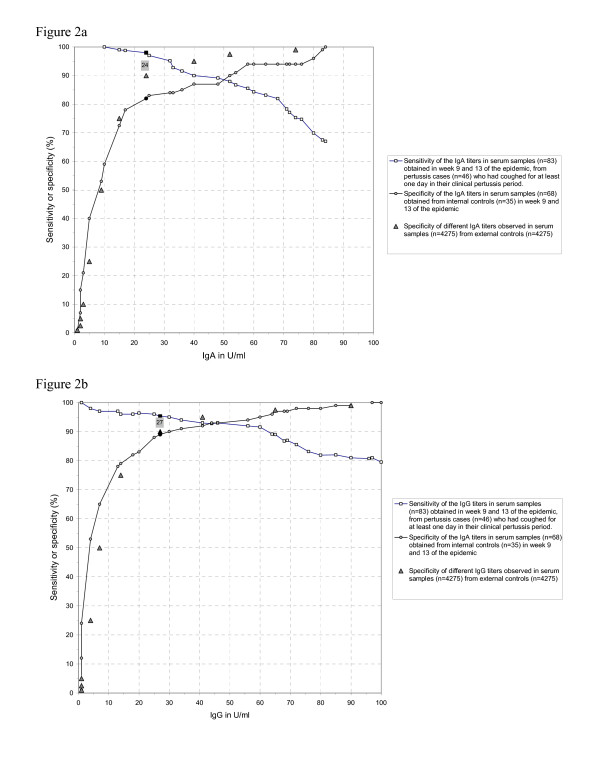
**Sensitivity and specificity of IgA and IgG for estimation of optimal cut-off levels**. Sensitivity and specificity of different IgA (figure 2a) and IgG (figure 2b) antibody levels (U/ml) against *B. pertussis *from the convent population during the pertussis epidemic. Sensitivities are calculated in the 83 serum samples obtained in week 9 and 13 of the epidemic, from the pertussis cases (n = 46) who had been coughing for between 1 and 87 days of their total period of clinical pertussis. Pertussis cases had a laboratory confirmed *B. pertussis *infection and a clinical pertussis (see methods). Specificities are calculated in the 68 serum samples obtained in week 9 and 13 of the epidemic from the internal controls (n = 35). Specificities of IgA and IgG levels calculated in the sera (n = 4275) of external controls (n = 4275) are indicated as well. The sensitivity en specificity mark in pertussis cases and internal controls of the IgA level of 24 U/ml and the IgG level of 27 U/ml with a specificity of 90% in external controls are indicated in black symbols.

Figure 2 shows the sensitivities of single IgA and IgG levels calculated in the 83 serum samples obtained in week 9 and 13 of the epidemic from the 46 pertussis cases who had been coughing for at least one day during their period of clinical pertussis.

Sensitivities of different levels of IgA (24–74 U/ml) and IgG (27 – 90 U/ml) ranged from 98% to 75% and 95% to 81%, respectively. The sensitivity of IgG was 80% at 100 U/ml and 71% at 200 U/ml (not shown in Figure 2). Compared to IgG, sensitivities of IgA started higher but decreased faster with increasing levels.

To probe spectrum bias[42] in the sensitivities obtained in our 46 pertussis cases, we also calculated sensitivities in the 91 IgA and 91 IgG levels obtained in weeks 9 and 13 of the epidemic of all 50 subjects with clinical pertussis, after they had coughed for at least 1 day during their period of clinical pertussis (Table 1). This resulted in lower sensitivities of IgA levels (24–74 U/ml) and IgG levels (27–90 U/ml) ranging from 90% to 69% and from 87% to 73%, respectively, while the sensitivity of IgG of 100 U/ml decreased from 80% to 72%. The lower sensitivities can be explained by the addition of concentrations from three subjects with clinical pertussis who had been coughing for 7, 11, and 21 days, respectively, and with IgA and IgG levels of at most 14 U/ml and 4 U/ml, respectively, suggesting that these three subjects did not have pertussis infection. The addition of a fourth subject increased the sensitivities. Indeed, this was most probably a missed pertussis case with 255 days clinical pertussis and highest obtained IgA and IgG levels of 116 and 80 U/ml, and a 3.6-fold decrease of IgG, while we used a 4-fold change in our case definition. A 3-fold change has been accepted as significant in a sufficiently precise assay.[43]

The specificities calculated in the 68 concentrations in serum samples obtained from the 35 internal controls in the convent population at weeks 9 and 13 of the epidemic are shown in Figure 2. Specificities of different levels of IgA (24–74 U/ml) and IgG (27–90 U/ml) levels ranged from 82% to 94% and 89% to 99%, respectively. The specificity of IgG was 100% at 100 U/ml. The specificities of different levels of IgA (24–74 U/ml) and IgG (27–90 U/ml) from 4275 sera samples from the 4275 external controls ranged from 90 to 99% (Figure 2). In the external controls IgA levels showed higher specificities than similar IgG levels.

To probe spectrum bias in the specificities obtained in our 35 internal controls, we calculated specificities as well in the 90 IgA and IgG concentrations obtained in weeks 9 and 13 of the epidemic of all 47 subjects with no cough (Table 1). This resulted in lower specificities of different levels of IgA (24–74 U/ml) and IgG (27–90 U/ml) ranging from 71% to 85% and 72% to 90%, respectively. The specificity of 100 U/ml IgG decreased from 100% to 92%. The lower observed IgA specificities were due to the high levels from the 12 added subjects with a laboratory confirmed pertussis infection and without a pre-epidemic cough or a clinical pertussis. Indeed, 15 of the 22 IgA levels from these 12 subjects were at least 24 U/ml, and of the IgG levels, 18 of 22 were at least 27 U/ml, and 8 were at least 100 U/ml. So the added 12 subjects were not a true control group of unexposed and uninfected subjects.

### Sensitivities at different time intervals

Table 3 shows the sensitivities of IgA (24–74 U/ml) and IgG (27–90 U/ml) levels calculated from the 46 pertussis cases (see Figure 2) at different time intervals in reference to the first day of coughing. In the first two weeks after the onset of clinical pertussis, cut-off levels of 24 U/ml for IgA and 27 U/ml for IgG showed sensitivities of 100% and 75%, respectively. In the third week of infection, a sensitivity of 100% was calculated for both parameters. From the fourth week onwards, the sensitivity remained at 97%.

**Table 3:** Specificities in external controls (n = 4275) and sensitivities in time intervals in reference to the onset of cough in pertussis cases (n = 46) of single IgA and IgG concentrations (see Figure 2). Geometrical Mean Concentrations (GMC) and number of samples are indicated.

**Level (U/ml)**^a^	**Specificity external controls**	**Sensitivity (%) in time intervals with reference to the onset of cough**				
		-21 to 0 days	1 to 14 days	15 to 21 days	22 to 87 days	1 to 87 days

**IgA**						
24	90.0%	33	100	100	97	98
40	95.0%	33	63	82	95	90
52	97.5%	17	63	82	92	88
74	99.0%	17	63	64	78	75

**GMC (U/ml)**		11	60	136	212	177

**IgG**						
27	90.0%	33	75	100	97	95
41	95.0%	33	75	91	95	93
65	97.5%	33	50	82	94	87
90	99.0%	33	38	82	86	81
100^b^	99–100%	33	38	82	84	80

**GMC (U/ml)**		8	48	390	506	391

**No. samples**		6	8	11	64	83

^a ^Levels are obtained from the estimation of optimal cut-off values in Figure 2.

^b ^Used by the National Institute for Public Health and the Environment in The Netherlands for surveillance.

### Waxing and waning of IgA and IgG between diagnostic parameters

It was estimated that IgA reached the 100% sensitivity and 99% specificity levels earlier than IgG. IgG waned more quickly back towards pre-infection levels. IgA and IgG remained above their 100% sensitivity level during 7.2 and 5.1 months respectively and above their 99%-specificity level during 5.5 and 4.3 months, respectively (Table 4). The rate of increase of IgA and IgG levels indicates that the paired samples should be collected within 4 and 7 days, respectively, after they begin to increase in order to observe the fourfold rise towards their GMC.

**Table 4:** Estimated waxing and waning of IgA and IgG levels between diagnostic parameters

**Variable**	**IgA**	**IgG**
Laboratory detection limit	5 U/ml	5 U/ml
100% sensitivity level in the 46 pertussis cases	24 U/ml	27 U/ml
90% specificity level in 4275 external controls	24 U/ml	27 U/ml
99% specificity level in 4275 external controls	74 U/ml	90 U/ml
GMC of the highest level obtained in week 9 and 12 of pertussis cases with at least 4-fold increase or decrease between week 9, 13 and 60 of the epidemic	229 U/ml (n = 28 levels)	406 U/ml (n = 49 levels)
Average speed of significant increase	16.0 U/ml/day (n = 11 level pairs)	14.7 U/ml/day (n = 9 level pairs)
Average speed of significant decrease	1.0 U/ml/day (n = 18 level pairs)	2.9 U/ml/day (n = 46 level pairs)
Mean time to increase from detection limit to 100% sensitivity level	1.2 days	1.5 days
Mean time to increase from detection limit to 99% specificity level	4.3 days	5.8 days
Mean time to increase from detection limit to GMC	14.0 days	27.3 days
Mean time to increase from 99% specificity level to GMC	9.8 days	21.5 days
Mean time to decrease from GMC to 99% specificity level	156.0 days	109.0 days
Mean time spent above 99% specificity level	165.8 days (5.5 month)	130.5 days (4.3 month)
Mean time to decrease from 99% specificity level to 100% sensitivity level	50.0 days	21.7 days
Total time spent going up and down between detection limit and GMC	238.0 days	165.6 days
Total time spent going up and down between 100% sensitivity level and GMC	217.8 days (7.2 months)	156.5 days (5.1 months)

## Discussion

Early diagnosis of pertussis in adults for outbreak management requires low cut-off levels for single IgA and IgG serological tests. We found that cut-offs of 24 U/ml for IgA and 27 U/ml for IgG led to a specificity of 90% and a sensitivity of 100% and 75%, respectively, during the first 2 weeks of pertussis. In the third week, the sensitivity was 100% for both tests. The sensitivity decreased slightly to 98% for IgA, and 95% for IgG during the first 87 days of clinical pertussis. After acute onset of pertussis, IgA remained above 24 U/ml for a mean duration of 7.2 months, and IgG remained above 27 U/ml for a mean duration of 5.1 months.

Although this study is limited by the relatively small number of subjects, the results are based on a pertussis epidemic in a defined community, with 100% participation. We are not aware of another study in which sensitivities of single IgA and IgG were evaluated in both the pre-clinical and clinical phases of pertussis.

The definition of pertussis was partly based on single IgA and IgG levels, which were also evaluated as diagnostic marker. This may have caused some incorporation bias.[42,44] However, 42 of the 46 pertussis cases were based on at least 4-fold changing IgA or IgG levels in paired samples (Table 1). The lowest IgG level of the 4 cases identified with a single IgG sample was 376 U/ml and they had coughed between 44 – 263 days. From 3 of these 4 cases we were not able to obtain a third serum sample in week 60 (2 subjects died earlier with pertussis), in order to detect significant change. The fourth subject showed a 3.6-fold changing IgG level. In addition, we did not find other causes of this epidemic of cough[12] in the ideal epidemic circumstances of a convent population with positive cultures for *B. pertussis*.

In our evaluation of spectrum bias in sensitivity and specificity, we showed that our choice of pertussis cases and controls was sound. Arguably, the use of our 35 internal controls may have led to underestimation of the specificity of single low IgA and IgG levels. Indeed 7 of the 35 internal controls had high IgA levels ranging from 24 – 74 U/ml and 2 internal controls had IgA levels above 74 U/ml. Six internal controls had IgG levels ranging from 27 – 90 U/ml and one internal control above 90 U/ml. We argue that IgA levels above 24 U/ml and IgG levels above 27 U/ml decreased the specificities obtained in the internal controls, compared with the specificities obtained in the external controls (Figure 2). Among all 47 subjects with no cough (Table 1), 21 (45%) had high single IgA and/or IgG levels. Because these 21 subjects with high single IgA and/or IgG levels had a serological indication of infection (antibody boosting) without symptoms, we consider the external control group, with GMC of IgA and IgG levels significantly lower than in the 46 pertussis cases, the better choice for calculating specificities.

The retrospective part of the study may have resulted in recall bias regarding onset of cough. The study however, was supported by a nun who was responsible for looking after all the nuns with pertussis during the epidemic. This registered nurse kept reliable clinical records including the history of coughing before and after the start of the study. We are therefore fairly confident that recall bias is limited.

IgA antibodies to *B. pertussis *antigens in whole-cell sonicate is known to lack specificity[45] compared to IgA antibodies to pertussis toxin.[30] Indeed, single high values of IgA and IgG antibodies to pertussis toxin indicate infections in adults, and IgA is more indicative of a recent antibody response, although less consistent than IgG.[30] However, we excluded causes of the epidemic other than pertussis and used culture, PCR, and IgG against pertussis toxin to positively identify pertussis cases. In our external control group, the levels of IgA against whole cell sonicate showed higher specificities than similar IgG levels. In the original Dutch study of IgA antibodies, it was stated that IgA antibodies, which are not induced by vaccination, can be used as a reliable indicator of natural infection with *B. pertussis *in adults within one week of infection,[38] especially if interpreted in connection with clinical findings.[46] On the other hand it has been postulated that because of the prolonged antibody response, IgA is not such a useful marker for recent infection.[38] We estimated however, that IgA reaches 100% sensitivity and 99% specificity level sooner than IgG, and that IgA and IgG remain above the 99% specificity level for a mean duration of 5.5 and 4.3 months, respectively, after the onset of pertussis.

IgG antibodies against the virulence factors pertussis toxin, pertactin, and fimbriae increase and decrease after both natural infection and vaccination.[31,47-49] In bacteriologically proven pertussis cases, IgG antibodies declined more rapidly than IgA.[31] This was confirmed in our study. IgG levels of at least 25 IU/ml were associated with *B. pertussis *infection in a previous study.[31] In our study, a cut-off level of 27 U/ml for IgG resulted in a sensitivity of 100% the third week after the onset of pertussis symptoms, and remained at 97% up to the 13^th ^week with a specificity of 90%.

We determined a sensitivity of 90% for 50 U/ml IgG and of 80% for 100 U/ml, which is comparable to a previous evaluation that determined a sensitivity of 89% for IgG levels above 50 U/ml and 76% for IgG levels of ≥ 100 U/ml.[35] Also, our specificity of 99% for an IgG level of 90 U/ml is comparable to that found in the prior study[35] which concluded that, independent of age, a cut-off level of 100 U/ml IgG showed a specificity of 99–100%. In the prior study, most patients reached IgG levels of 100 U/ml within 4 weeks of disease onset which persisted for 4.5 months. These findings are in line with our estimates that the IgG level increases from the detection limit (5 U/ml) to 100 U/ml in 6.7 days and persists at this level for 4.2 months. The rate of IgA and IgG increase underlines the importance of obtaining acute phase samples early in the disease in order to detect a significant increase, and consequently the importance of significantly decreasing levels for ultimately diagnosing pertussis if the first sample is not obtained early in the disease.[33,34] Our outcomes are also in line with a study in which a cut-off point of 94 IU/ml for IgG pertussis toxin, with a sensitivity of 80% and a specificity of 93%, has been proposed.[50] This 94 IU/ml is comparable with 76 U/ml in our study and considerable lower then the 125 IU/ml (= 100 U/ml) officially used in the Netherlands. In our study 94 IU IgG pertussis toxin had a sensitivity of 83% and a specificity of 98%.

Our findings for low IgA and IgG levels to diagnose pertussis in outbreak management are supported by findings from a pertussis outbreak in a boarding school in Australia, where IgA against whole-cell sonicate, and IgG against pertussis toxin proved useful for early diagnosis and outbreak management. In that study, it was concluded that the IgG level of 125 IU/ml (100 U/ml) was not sensitive enough to identify pertussis cases in their early stages for outbreak management.[51]

Because IgA is not induced by vaccination against pertussis, it may be preferred over IgG in recently vaccinated subjects, as IgG is induced by vaccination with whole-cell vaccines against pertussis used in the Netherlands.[31] Other vaccines may induce even higher IgG-pertussis toxin levels, since the response to pertussis toxin varies between different whole-cell vaccines and acellular vaccines. These IgG-pertussis toxin levels can reach levels higher than 100 U/ml.[48,52-54]

## Conclusion

High sensitivity and specificity are required to track and exclude pertussis in vaccine efficacy trials[43] if another serious disease is suspected, and in passive surveillance systems used to estimate vaccine efficacy. In clinical practice and outbreak situations, diagnosis of *B. pertussis *illness must be immediate to allow for prompt therapeutic intervention to reduce disease severity and spread. Therefore, diagnostic criteria should be sensitive, even if specificity is compromised.[55] Pertussis has recognizable characteristic clinical symptoms to a physician.[56] A clinical case definition can be used in clinical practice and outbreak management,[57,58] and for antibiotic management.[59] We conclude that after infection with *B. pertussis *IgA and IgG concentrations start to increase from low levels upwards, and that low cut-off levels of 24 U/ml for IgA antibodies and 27 U/ml (equivalent to 27 IU/ml) for IgG antibodies could be considered as practical tools for laboratory confirmation of clinical pertussis in adults during the first 3 weeks of disease outbreak for public health practice, e.g. outbreak management. We suggest a more extensive study of the value of IgA antibodies to pertussis toxin for the early diagnosis of pertussis.

## Competing interests

The author(s) declare that they have no competing interests.

## Authors' contributions

PLJMM conceived the study, performed the analysis and drafted the manuscript. FSS assisted with the analysis of microbiological data and helped draft the manuscript. EWS assisted with the statistical analysis and helped draft the manuscript. JHR participated in the design and coordination of the study and helped draft the manuscript. All authors read and approved the final manuscript.

## Pre-publication history

The pre-publication history for this paper can be accessed here:


